# Esophageal endoscopic muscularis dissection with a tunnel-first approach for deeply invasive Barrett’s adenocarcinoma

**DOI:** 10.1055/a-2880-0234

**Published:** 2026-06-11

**Authors:** Giorgi Tvaradze, Mayo Tanabe, Kazuki Yamamoto, Stefan Groth, Ewerton Marques Maggio, Haruhiro Inoue, Stefan Seewald

**Affiliations:** 1 Center for Gastroenterology 30364 Klinik HirslandenZürichZHSwitzerland; 2 Digestive Diseases Center 378609 Showa Medical University Koto Toyosu HospitalKotoTokyoJapan; 3 medica MEDICAL LABORATORIES Dr. F. KAEPPELI AGZürichSwitzerland

In selected cases of deep submucosal esophageal cancer, complete endoscopic resection may be appropriate when surgery is not feasible. In such situations, endoscopic muscularis dissection (EMD) may be required to achieve an R0 resection, 1​ as dissection can extend into the superficial circular muscle layer. The aim of this approach is to enable complete tumor removal despite deep submucosal invasion while preserving esophageal wall integrity and avoiding perforation. A tunnel-first approach adds an important safety advantage to EMD by allowing the precise assessment of both the depth and the longitudinal extent of tumor infiltration, while at the same time providing a stable and protected working space for muscularis dissection. 2​

**Fig. 1:**
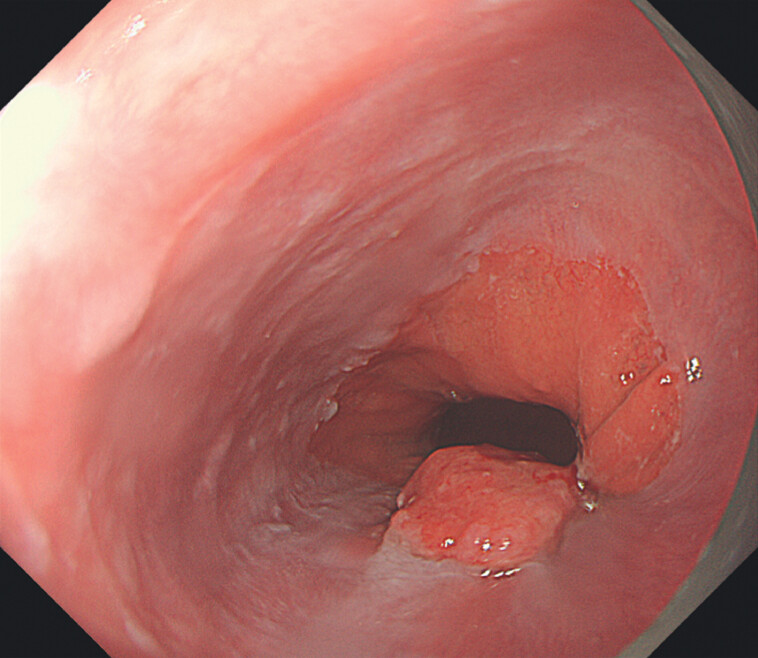
An endoscopic view of a 1.5 cm Barrett adenocarcinoma with suspected deep submucosal invasion (Paris classification—Is).

A 45-year-old man with metastatic esophageal squamous cell carcinoma undergoing systemic therapy presented with a newly detected 1.5 cm Barrett’s adenocarcinoma located at the *Z* -line ( Fig. 1 ). Pre-procedural imaging and endoscopic ultrasound suggested deep submucosal infiltration, and endoscopic resection was pursued as palliative local treatment.

**Fig. 2:**
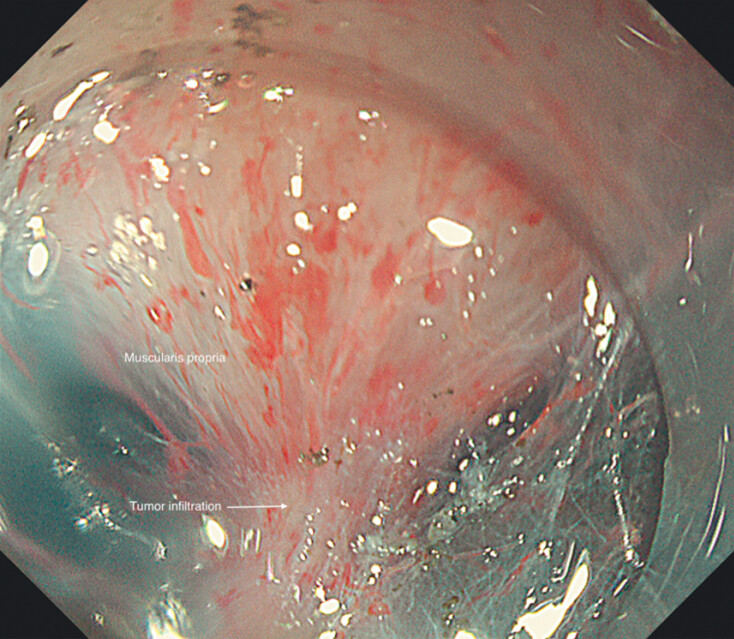
An endoscopic view within the submucosal tunnel demonstrating the direct visualization of tumor infiltration in relation to the muscular layer.

**Fig. 3:**
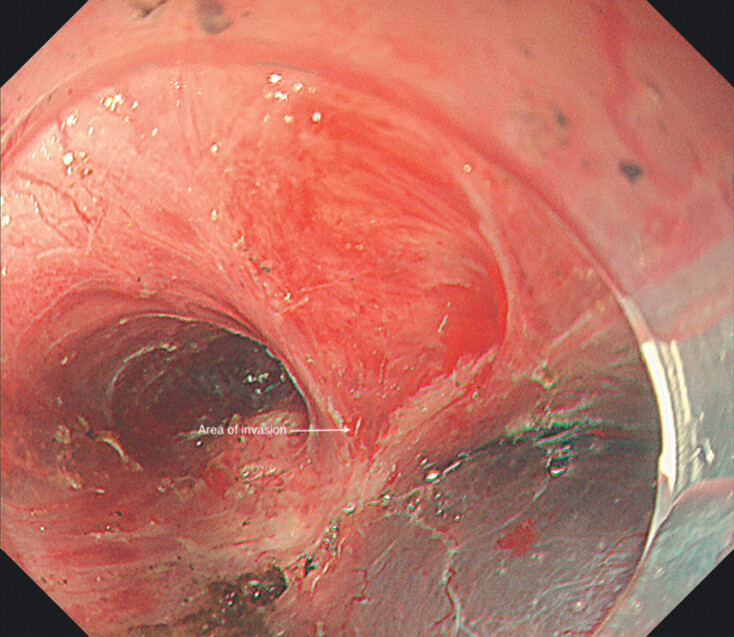
Dissection within the tunnel extended laterally on the left side of the invasion area to assess the longitudinal extent of tumor infiltration and define the dissection plane.

**Fig. 4:**
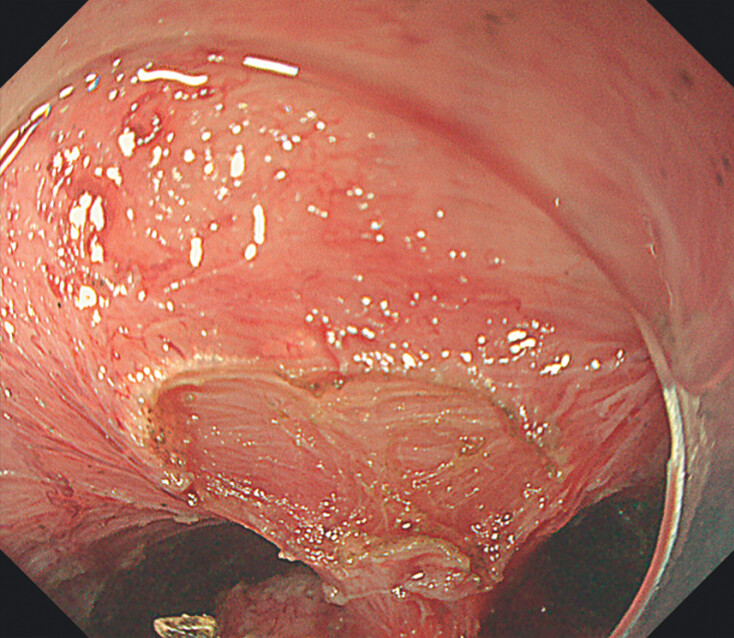
An early stage of endoscopic muscularis dissection (EMD) showing controlled dissection of the superficial circular muscle layer under direct endoscopic visualization.

This case demonstrates the safe application of EMD in the esophagus using a tunnel-first approach ( Video 1 ). After the creation of the submucosal tunnel, the area of invasion became clearly identifiable ( Fig. 2 ), and lateral dissection on both sides defined the depth and longitudinal extent of infiltration ( Fig. 3 ). Within the tunnel, a clear muscular traction sign indicated superficial invasion into the muscle layer. Based on these findings, EMD was initiated with the careful dissection of the superficial circular muscle layer ( Fig. 4 ). After central dissection within the tunnel, the specimen was released by intraluminal dissection of the remaining circumferential margins.

**Video 1** Endoscopic muscularis dissection using a tunnel-first approach for deeply invasive Barrett’s adenocarcinomas, enabling the precise assessment of invasion depth and safe local resection.


**Fig. 5:**
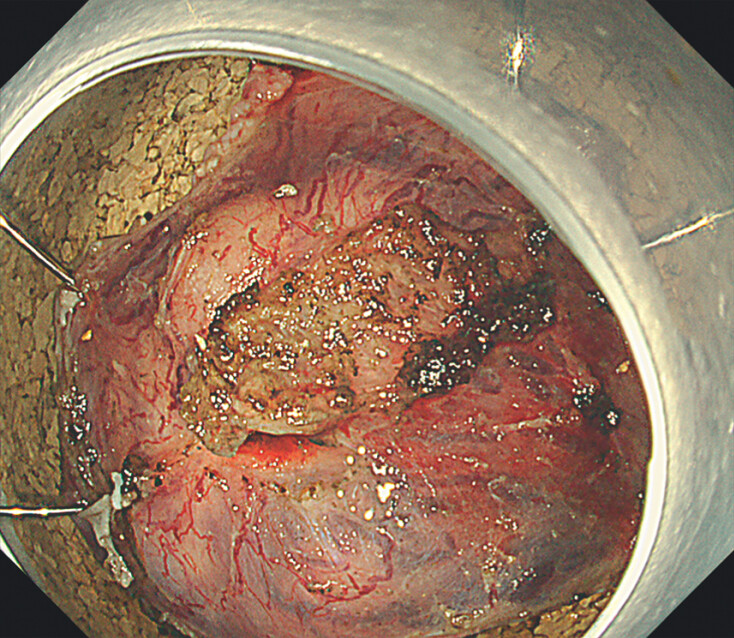
The resected specimen was flipped to reveal the muscularis layer at the site of tumor invasion.

An en bloc R0 resection was achieved without complications ( Fig. 5 ). Histopathology confirmed an adenocarcinoma with sm2 invasion and lymphatic invasion.

This case highlights that tunnel-first EMD enables the accurate intra-procedural assessment of invasion depth and facilitates a safe, controlled, and oncologically adequate resection. Even in non-surgical or palliative settings, it may allow complete local tumor removal while preserving esophageal integrity. 3​ To our knowledge, the use of a tunnel-first EMD strategy for deeply invasive Barrett’s adenocarcinomas has not been previously described.

Endoscopy_UCTN_Code_TTT_1AO_2AG_3AZ
